# The Effects of a Transgelin-2 Agonist Administered at Different Times in a Mouse Model of Airway Hyperresponsiveness

**DOI:** 10.3389/fphar.2022.873612

**Published:** 2022-06-16

**Authors:** Hong-Kai Yuan, Jin Lu, Xue-Ling Wang, Zhi-Ying Lv, Bo Li, Weiliang Zhu, Yong-Qing Yang, Lei-Miao Yin

**Affiliations:** ^1^ Yueyang Hospital, Shanghai University of Traditional Chinese Medicine, Shanghai, China; ^2^ CAS Key Laboratory of Receptor Research, State Key Laboratory of Drug Research, Drug Discovery and Design Center, Shanghai Institute of Materia Medica, Chinese Academy of Sciences, Shanghai, China

**Keywords:** transgelin-2, agonist, TSG12, administration time, asthma, airway hyperresponsiveness

## Abstract

Airway hyperresponsiveness (AHR) is one of the most important features of asthma. Our previous study showed that inhaled transgelin-2 agonist, TSG12, effectively reduced pulmonary resistance in a mouse model of asthma in a dose-dependent manner. However, the optimal administration time of TSG12 to reduce AHR and the pharmacological effects are still unclear. In this study, the effects of TSG12 inhalation before and during AHR occurrence were examined. The results showed that the pulmonary resistance was reduced by 57% and the dynamic compliance was increased by 46% in the TSG12 Mch group (atomize TSG12 10 min before methacholine, *p* < 0.05 vs. model). The pulmonary resistance was reduced by 61% and the dynamic compliance was increased by 47% in the TSG12 + Mch group (atomize TSG12 and methacholine together, *p* < 0.05 vs. model). Quantitative real-time PCR showed that the gene expression levels of transgelin-2, myosin phosphatase target subunit-1, and myosin light chain were up-regulated by 6.4-, 1.9-, and 2.8-fold, respectively, in the TSG12 Mch group. The gene expression levels of transgelin-2, myosin phosphatase target subunit-1, and myosin light chain were up-regulated by 3.2-, 1.4-, and 1.9-fold, respectively, in the TSG12 + Mch group. The results suggested that TSG12 effectively reduces pulmonary resistance when TSG12 inhalation occurred both before and during AHR occurrence. Gene expression levels of transgelin-2 and myosin light chain were significantly up-regulated when TSG12 inhalation occurred before AHR occurrence. This study may provide a basis for the administration time of TSG12 for asthma treatment in the future.

## Introduction

Asthma is one of the most common chronic respiratory diseases worldwide and is characterized by airway hyperresponsiveness (AHR) (Cockcroft et al., 2020). More than 339 million people worldwide suffer from asthma, which is expected to increase to 400 million by 2025 (Barcik et al., 2020). The prevalence of asthma varies from 1 to 18% in different regions (White et al., 2018). In China, the total prevalence of asthma is 4.2% (Huang et al., 2019). Based on the 2020 Chinese population, the number of Chinese patients with asthma was approximately 61 million (National Bureau of Statistics of China, 2021). In addition, asthma imposes a severe economic burden (Wenzel, 2012). The annual direct cost of asthma is 82 billion dollars in America and more than 72 billion euros in Europe (Forno and Celedon, 2019; Asthma and Allergy Foundation of America, 2021). Therefore, asthma has become a serious challenge in modern clinics.

The primary goal of asthma treatment is to reverse early airway changes, limit the late effects of airway remodeling to effectively control AHR, and improve the quality of life of patients (Choby and Lee, 2015). However, asthma patients still have recurrent symptoms despite standard treatments (Huang and Pansare, 2019). It has been reported that only 45% of patients have an effective response to standard asthma medications (Peters et al., 2007). Therefore, many new treatments have been developed to meet the needs of asthma patients (Cevhertas et al., 2020). For example, tezepelumab is a new human monoclonal antibody drug that can reduce the exacerbation rate of asthma by blocking thymic stromal lymphopoietin (Taleb and Badgett, 2021). The clinical trial showed that the annualized exacerbation rate of asthma was twice as high with placebo than that with tezepelumab among patients with uncontrolled asthma (Menzies-Gow et al., 2021). Carvacrol is a peroxisome proliferator–activated receptor-alpha agonist that effectively inhibits airway inflammation in asthmatic patients by reducing the release of asthma-related inflammatory mediators (Gholamnezhad et al., 2016). The inflammatory markers IL-4, IL-5, and IL-13 were significantly reduced by 49, 52, and 28%, respectively, compared to asthma rats after carvacrol (15 mg/kg) treatment (Ezz-Eldin et al., 2020). In addition, clinical trials also showed that treatment with carvacrol (1.2 mg/kg) for 2-month also significantly decreased IL-4 levels in serum (Ghorani et al., 2021). Although these new drugs bring possibilities for asthma treatment, new therapies are still needed to give patients with uncontrolled asthma more choice.

Our previous studies showed that transgelin-2 is a new therapeutic target for asthma (Yin et al., 2018; Yin et al., 2019). Transgelin-2 receptor activation has been shown to regulate airway smooth muscle relaxation by affecting the phosphorylation level of key proteins in the calcium sensitization pathway (Meng et al., 2017). A specific transgelin-2 agonist, TSG12 (PubChem CID: 1896138, molecular formula: C_21_H_21_N_3_O_4_S_3_, IUPAC name: N-[4-[(8-methoxy-4, 4-dimethyl-5H-dithiolo [3, 4-c] quinolin-1-ylidene) amino] phenyl] sulfonyl acetamide), can effectively reduce AHR (Yin et al., 2018). Our previous studies have preliminarily shown the efficacy of TSG12 in cellular and asthma animal models. TSG12 inhibited the contraction of rat airway smooth muscle cells induced by acetylcholine in a dose-dependent manner, and the half-maximum effective concentration (EC_50_) was 6.8 nM. We analyzed the role of TSG12 in two asthma models, including mice challenged with ovalbumin or house dust mite. The inhalation of TSG12 significantly reduced pulmonary resistance in ovalbumin-induced mice. *In vivo*, TSG12 reduced pulmonary resistance more effectively than conventional therapy with β_2_-agonist terbutaline. Similarly, TSG12 reduced the pulmonary resistance by more than 80% in the mice model of asthma induced by house dust mite and TSG12 was more effective than conventional therapy with β_2_-agonist isoproterenol in reducing pulmonary resistance. In addition, we had confirmed that TSG12 induced the dephosphorylation of myosin phosphatase target subunit-1 (MYPT1) and myosin light chain (MLC) in airway smooth muscle cells. Unphosphorylated MYPT1 at threonine 853 activated the myosin light chain phosphatase complex to dephosphorylate MLC and induced muscle relaxation (Yin et al., 2018). These findings suggest that TSG12 has a good clinical application prospect in treating the AHR of asthma (Crunkhorn, 2018).

However, no studies have been performed to evaluate the optimal administration time of TSG12 and related pharmacological effects. In this study, the effects of TSG12 inhalation at two different administration times, before and during AHR occurrence, on pulmonary functions in mice were examined. The changes in pulmonary resistance and dynamic compliance were studied in mice using the resistance and compliance system. Hematoxylin–eosin staining was used to study the effect of different administration time on inflammatory changes in lung tissues and quantitative real-time polymerase chain reaction (qRT-PCR) was used to study the effect of different administration time on related gene expression.

## Materials and Methods

### Animals

Male C57BL/6J mice (6 weeks old, weighing 18.1 ± 2.9 g) were purchased from Beijing Vital River Laboratory Animal Technology Co. Ltd. The animals were housed under normal laboratory conditions (temperature at 23 ± 1°C, humidity at 55 ± 5%). The mice had free access to food and water under a 12-h light and dark cycle. All experimental protocols were approved by the Shanghai University of Traditional Chinese Medicine for Experimentation on Animals.

### Drugs and Treatment Preparation

Methacholine (Mch, Sigma-Aldrich, Shanghai, China) was dissolved in normal saline. Terbutaline (TB, Huayu Pharmaceutical Co. Ltd., Chengdu, China) was diluted with normal saline. TSG12 (CAS Key Laboratory of Receptor Research, State Key Laboratory of Drug Research, Shanghai Institute of Materia Medica, Chinese Academy of Sciences) was dissolved in dimethyl sulfoxide (DMSO, Beyotime Biotechnology, Shanghai, China) and diluted with normal saline. The final concentration of DMSO is 0.02% with no effect on airway smooth muscle cells (Supplementary Figure S1). The solutions were prepared immediately before use.

### Animal Groups and Experimental Processes

**FIGURE 1:**
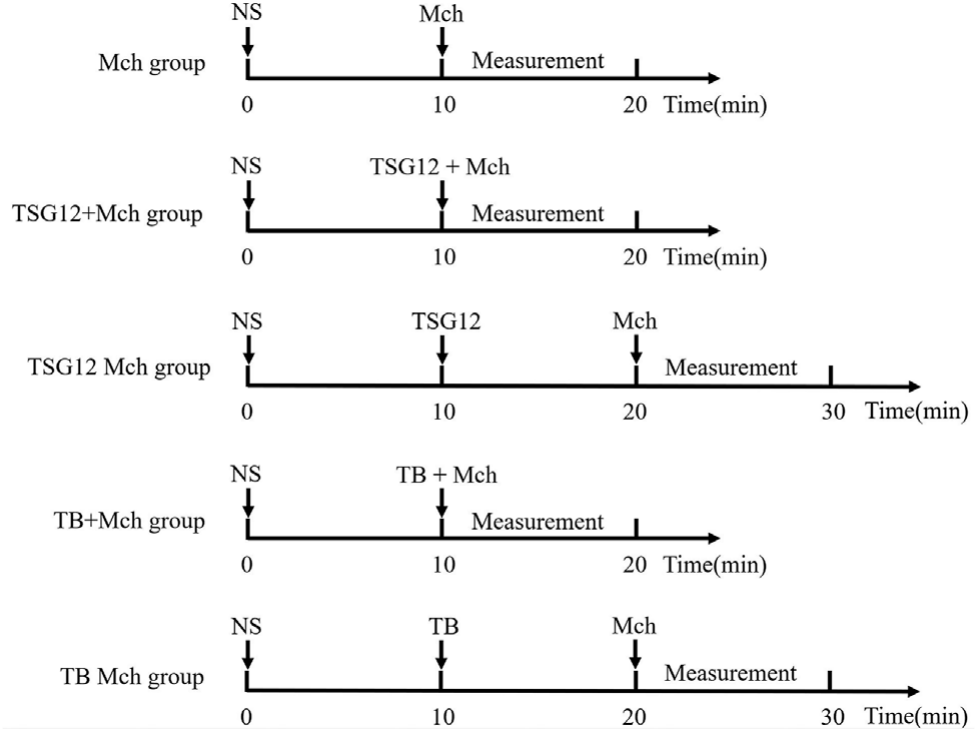
Schematic of administration. The Mch group was administered with atomized NS at 0 min and Mch at 10 min. The TSG12 + Mch group was atomized with NS at 0 min and TSG12 together with Mch at 10 min. The TSG12 Mch group was atomized with NS at 0 min, TSG12 at 10 min, and Mch at 20 min. The TB + Mch group was atomized with NS at 0 min and TB together with Mch at 10 min. The TB Mch group was atomized at 0 min, TB at 10 min, and Mch at 20 min. Abbreviations: NS, normal saline; Mch, methacholine; TB, terbutaline.

The mice were randomly divided into different groups (6 mice per group): blank group (atomize normal saline), Mch group (atomize Mch), TSG12 + Mch group (atomize TSG12 and Mch together), TSG12 Mch group (atomize TSG12 10 min before Mch), TB + Mch group (atomize TB and Mch together), and TB Mch group (atomize TB 10 min before Mch). As a normal control, normal saline was atomized for 10 min first in all groups. In the Mch group, the mice were challenged with Mch (12 mg/ml) to induce AHR. In the TSG12 + Mch group, TSG12 (100 ng/kg) and Mch (12 mg/ml) were administered together by aerosol. In the TSG12 Mch group, TSG12 (100 ng/kg) was administered by aerosol 10 min before Mch (12 mg/ml) inhalation. In the TB + Mch group, TB (100 μg/kg) and Mch (12 mg/ml) were administered together by aerosol. In the TB Mch group, TB (100 μg/kg) was administered by aerosol 10 min before Mch (12 mg/ml) inhalation. The dose ranges were selected based on previous studies (Yin et al., 2018). The schematic is shown in Figure 1.

### Pulmonary Function Measurement

Intrusive pulmonary function measurement *in vivo* can obtain sensitive and accurate pulmonary function parameters, and this method is convenient for drug administration through the airway (Hatchwell et al., 2015). Changes in pulmonary functions were studied in mice using the resistance and compliance system (DSI, Buxco RC). The anesthetic solution (1% pentobarbital sodium, 100 mg/kg) was injected intraperitoneally. After anesthesia, the mouse was fixed on the operating table and an incision was made in the trachea. Tracheal intubation was carefully performed, and the mouse was placed on the mouse table (Supplementary Figure S2). After the thoracic fluctuations of the mouse were consistent with the set respiratory frequency, normal saline was added into the atomizer to record the baseline, then Mch and drug were added separately. Pulmonary resistance (RL) and dynamic compliance (Cdyn) were recorded after the thoracic fluctuations of the mouse were consistent with the set respiratory frequency. All steps were performed according to instructions.

### Histological Analysis of Lung Tissue

The fresh left lobes of the lungs were fixed in 4% formaldehyde for 48 h and embedded in paraffin. Each paraffin-embedded lung block was cut into 4 μm thin slices and stained with hematoxylin–eosin (Beyotime Biotechnology, Shanghai, China). The hematoxylin–eosin staining was used to assess peribronchial inflammation. The inflammatory changes were assessed by the following score: 0, normal; 1, few cells; 2, a ring of inflammatory cells with 1 cell layer deep; 3, a ring of inflammatory cells with two to 4 cells deep; 4, a ring of inflammatory cells with more than 4 cells deep (Xue et al., 2021).

### Quantitative Real-Time Polymerase Chain Reaction (qRT-PCR)

To assess the gene expression of transgelin-2, MYPT1 and MLC by qRT-PCR, total RNA was extracted from lung tissue samples using the Trizol reagent. The RevertAid First Strand cDNA Synthesis Kit (Thermo Scientific, Shanghai, China) was used to convert total RNA into complementary DNA. Glyceraldehyde-3-phosphate dehydrogenase (GAPDH) was selected as control. The primers for each gene are listed in Table 1. The results of qRT-PCR were analyzed according to the 2^−ΔΔCt^ method (Zhou CY. et al., 2020). Briefly, the gene expression was calculated according to formula 2^(Rt−Et)^. Rt is the threshold cycle number for the reference gene (GAPDH) and Et is the threshold cycle number for the target genes (Transgelin-2, MYPT1, and MLC). The accumulated mRNA expression levels among different groups were compared and data were represented by the fold change. The changes with a 2-fold increase in the expression are considered to be up-regulated, and changes with a 0.5-fold decrease in the expression are considered to be down-regulated.

**TABLE 1:** Primer sequences used in qRT-PCR studies.

mRNA	Primer Sequence
GAPDH	Forward: 5′-GAT​GGA​CAC​ATT​GGG​GTT-3′
	Reverse: 5’-AAA​GCT​GTG​GCG​TGA​TG-3′
Transgelin-2	Forward: 5′-GCA​GGC​CCC​AGT​AAA​GA-3′
	Reverse: 5’-GGA​AGA​TGT​CCG​TGG​TGT-3′
MYPT1	Forward: 5′-TGG​AGG​TTA​GGG​CTT​CG-3′
	Reverse: 5′-GGG​ATT​GTA​GGG​GTG​ACA​T-3′
MLC	Forward: 5′-CAG​GGA​TCA​TGT​AGG​AGC​A-3′
	Reverse: 5′CGC​ACA​TAG​GCA​AGC​AA-3′

GAPDH, glyceraldehyde-3-phosphate dehydrogenase; MYPT1, myosin phosphatase target subunit 1; MLC, myosin light chain

### Statistics Analysis

The results are presented as the mean ± SEM (Standard Error of Mean). Statistical analysis was performed using IBM SPSS Statistics 24.0 (SPSS Inc, Chicago, IL, United States). Statistical significance among different groups was calculated using one-way ANOVA followed by the least significant difference (LSD) or the Games–Howell *post hoc* test based on the data and tested hypotheses. *p* values less than 0.05 was considered statistically significant.

## Results

### AHR Was Successfully Induced in Mice

**FIGURE 2:**
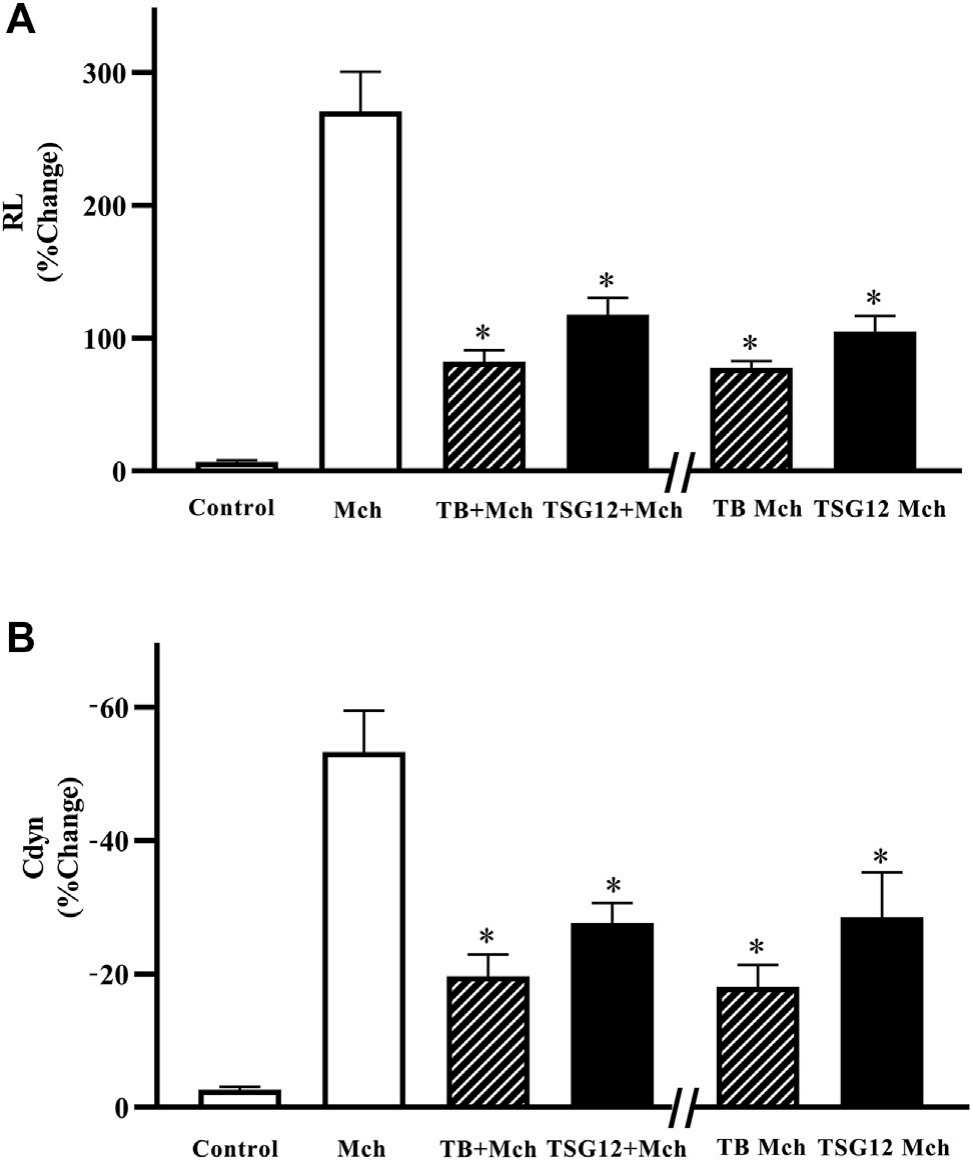
Changes in pulmonary resistance and dynamic compliance in mice with AHR. **(A)** RL increased 40-fold after stimulation with Mch (vs. blank). RL was reduced by 57% in the TSG12 Mch group and reduced by 61% in the TSG12 + Mch group (vs. Mch). **(B)** Cdyn was reduced 14-fold after stimulation with Mch (vs. blank). Cdyn was increased by 46% in the TSG12 Mch group and increased by 47% in the TSG12 + Mch group (vs. Mch). Data were presented as the mean ± SEM, N = 6, P<0.05 was considered statistically significant. Abbreviations: Cdyn, dynamic compliance; Mch, methacholine; RL, pulmonary resistance; SEM, standard error of mean.

After anesthesia, the mouse was first inhaled with normal saline as baseline, and Mch (12 mg/ml) was inhaled to induce AHR. Pulmonary function measurement showed that RL was increased by 40-fold (*p* < 0.05 vs. blank group, Figure 2A) and Cdyn was reduced by 14-fold after Mch-atomized (*p* < 0.05 vs. blank group, Figure 2B), showing that the mouse model of AHR was successfully established.

### TSG12 Reduced RL When Administered Both Before and During AHR Occurrence

The effect of TSG12 was studied on the mouse model of AHR and TB was selected as the positive control. TSG12 and TB were administered at two time points: 10 min before Mch or together with Mch. TSG12 reduced RL by 57% in the TSG12 Mch group and by 61% in the TSG12 + Mch group (*p* < 0.05 vs. Mch group, Figure 2A). TB reduced RL by 69% in the TB Mch group and by 71% in the TB + Mch group (*p* < 0.05 vs Mch group, Figure 2A). TSG12 can effectively reduce pulmonary resistance at 1/1000 doses (100 ng/kg) compared with TB (100 μg/kg) and there was no significant difference between the groups treated 10 min before AHR and together with AHR, showing that TSG12 can both effectively inhibit RL before and during AHR occurrence.

### TSG12 Increased Cdyn When Administered Both Before and During AHR Occurrence

TSG12 increased Cdyn by 46% in the TSG12 Mch group and by 47% in the TSG12 + Mch group (*p* < 0.05 vs. Mch group, Figure 2B). TB increased Cdyn by 63% in the TB Mch group and by 66% in the TB + Mch group (*p* < 0.05 vs. Mch group, Figure 2B). TSG12 can effectively increase Cdyn at 1/1000 doses (100 ng/kg) compared with TB (100 μg/kg) and there was no significant difference between the groups treated 10 min before AHR and together with AHR, showing that TSG12 can both effectively improve Cdyn before and during AHR occurrence.

### No Histopathological Changes Were Observed in Lung Tissues Among Different Groups

**FIGURE 3:**
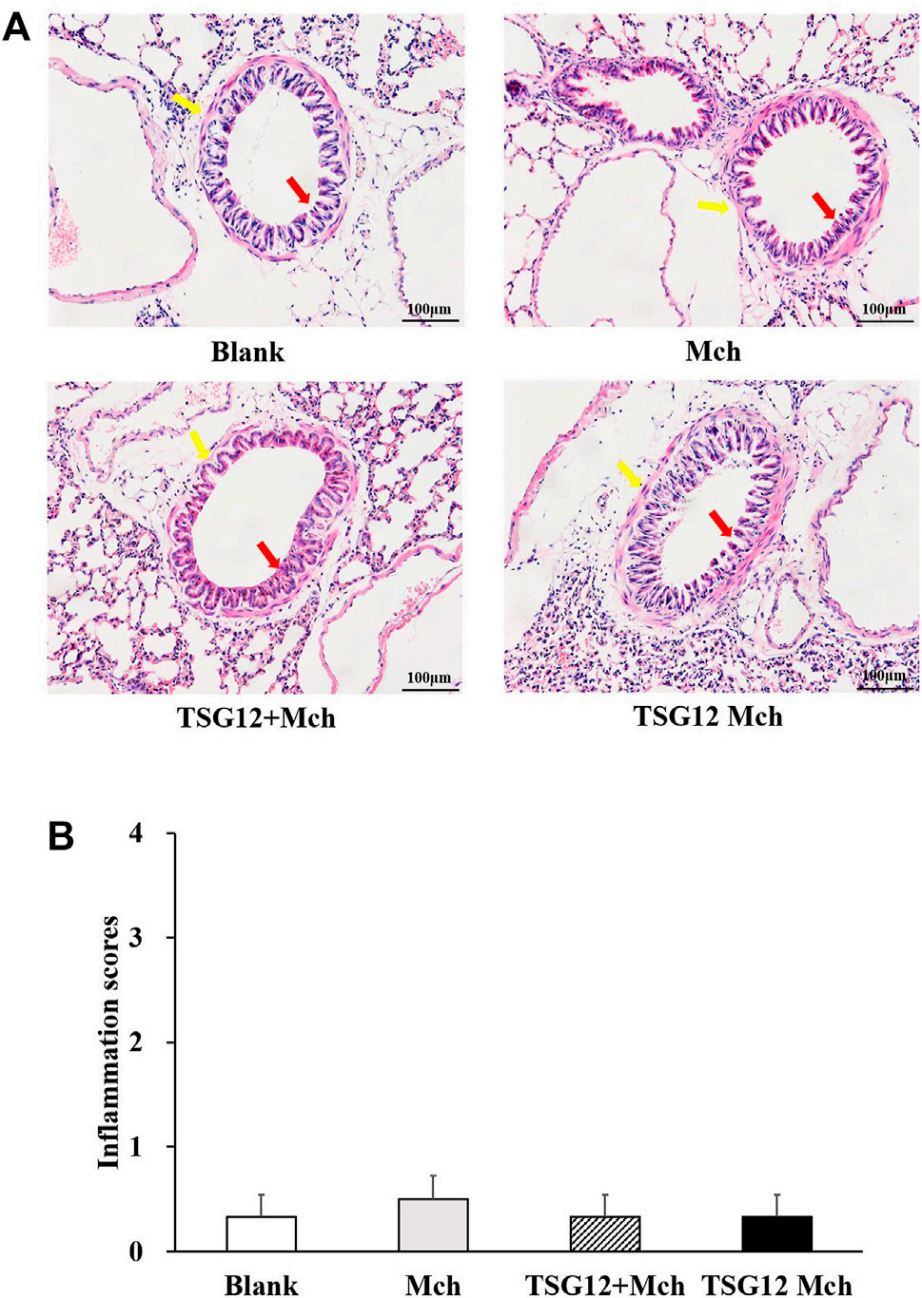
Histopathological lung changes among different groups. **(A)** In the blank group, the bronchiolar structure (red arrow) was clear and there was no inflammatory cell infiltration around the bronchiolar (blue arrow). In the Mch group, TSG12 + Mch group, and TSG12 Mch group, there were no significant changes compared with the blank group. **(B)** Inflammation scores among different groups. Data were presented as the mean ± SEM, N = 6, P<0.05 was considered statistically significant. Abbreviations: Mch, methacholine; SEM, standard error of mean.

Hematoxylin–eosin staining was used to measure the changes in inflammation among different lung tissues. In the blank group, the bronchiolar structure was clear and there was no inflammatory cell infiltration around the bronchiolar (Figure 3A). In the Mch group, there was no change in the structure of bronchiolar and no inflammatory cell infiltration around bronchi was found after Mch inhalation (vs. blank group, Figure 3B), showing that the transient AHR model does not lead to structural changes and inflammation in lung tissue. Likewise, the results showed normal bronchiolar structure with no inflammatory cell infiltration around bronchiolar in the TSG12 Mch group and TSG12 + Mch group (vs. Mch group, Figure 3A). There were no significant differences in inflammation scores among different groups (Figure 3B).

### The Expression of Transgelin-2 Changed Twice as Much in the TSG12 Mch Group as in the TSG12 + Mch Group

**FIGURE 4:**
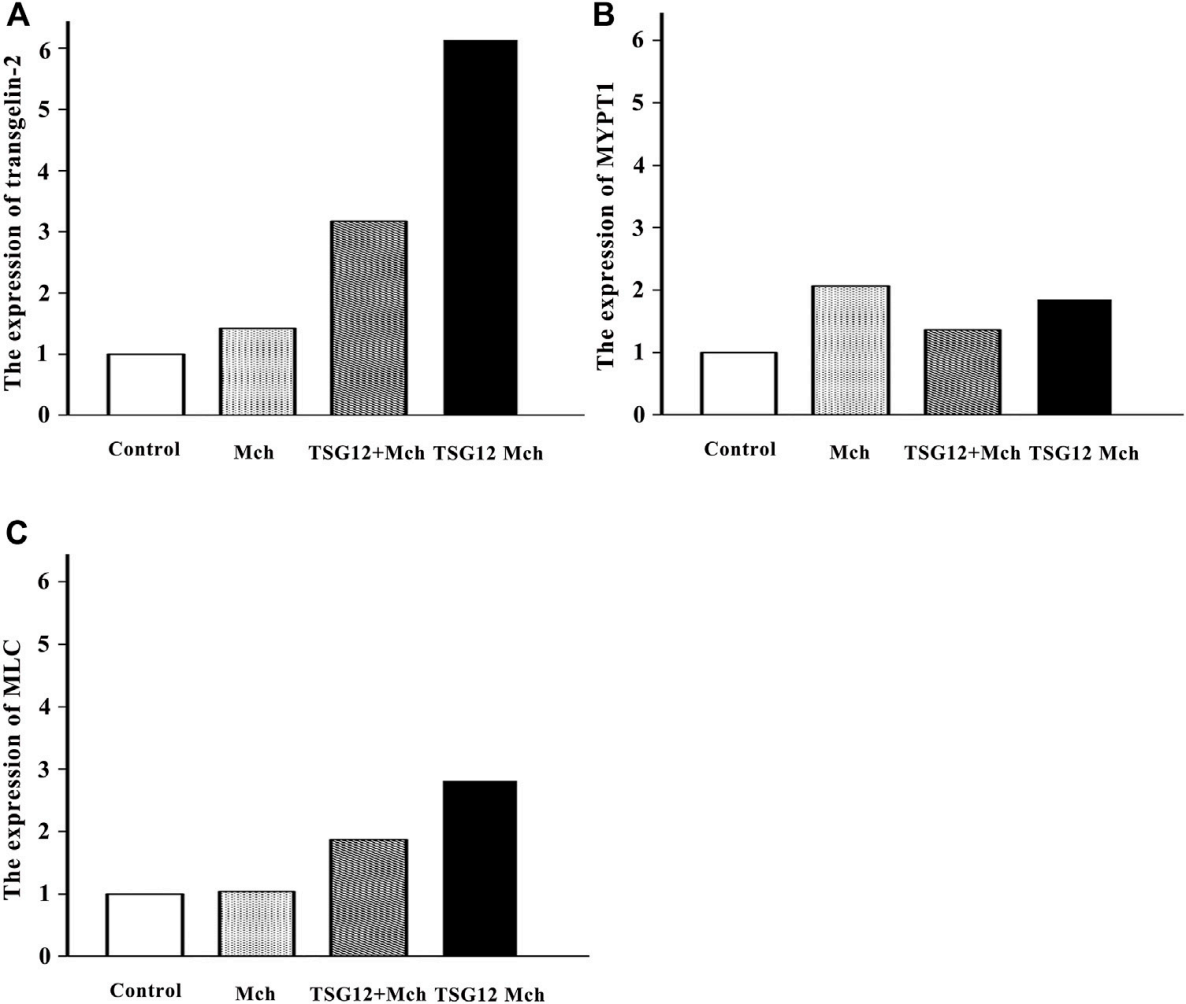
Expression of relevant genes after TSG12 administration. **(A)** Expression levels of transgelin-2 were up-regulated by 1.4-, 3.2-, and 6.1-fold in the Mch group, TSG12 + Mch group, and TSG12 Mch group, respectively (vs. blank). **(B)** Expression levels of MYPT1 were up-regulated by 2.1-, 1.4-, and 1.9-fold in the Mch group, TSG12 + Mch group, and TSG12 Mch group, respectively (vs. blank). **(C)** Expression levels of MLC were up-regulated by 1.1-, 1.9-, and 2.8-fold in the Mch group, TSG12 + Mch group, and TSG12 Mch group, respectively (vs. blank). Values were presented by the 2^−ΔΔCt^ method, N = 6, changes with a 2-fold increase in expression were considered to be up-regulated, and changes with a 0.5-fold decrease in expression were considered to be down-regulated. Abbreviations: Mch, methacholine; MYPT1, myosin phosphatase target subunit-1; MLC, myosin light chain.

The changes in the gene expression in transgelin-2, MYPT1, and MLC after TSG12 administration were studied by qRT-PCR. The expression levels of transgelin-2 were up-regulated by 1.4-, 3.2-, and 6.4-fold in the Mch group, TSG12 + Mch group, and TSG12 Mch group, respectively (vs blank group, Figure 4A). The expression levels of MYPT1 were up-regulated by 2.1-, 1.4-, and 1.9-fold in the Mch group, TSG12 + Mch group, and TSG12 Mch group, respectively (vs blank group, Figure 4B). Likewise, the expression levels of MLC were up-regulated by 1.1-, 1.9-, and 2.8-fold in the Mch group, TSG12 + Mch group, and TSG12 Mch group, respectively (vs blank group, Figure 4C).

## Discussion

The study first showed that the inhalation of TSG12 significantly reduced pulmonary resistance before Mch administration. This result is consistent with our previous results in asthmatic mice (Yin et al., 2018). Meanwhile, TSG12 inhalation with Mch also significantly reduced pulmonary resistance. Based on these data, our results show that TSG12 has not only a preventive effect but also have therapeutic effects for ameliorating the AHR of asthma. This finding provides a new clue for the pharmacological function and potential clinical applications of transgelin-2 agonist, such as TSG12.

### TSG12 Has Both Preventive Effect and Therapeutic Effect for Ameliorating AHR

One of the possible explanations for both preventive and therapeutic effects of TSG12 in reducing pulmonary resistance may be due to the rapid phosphorylation of transgelin-2 downstream proteins. Transgelin-2 is a unique actin-binding protein, which controls cellular functions such as cell contraction by regulating membrane–cytoskeleton interactions (Yin et al., 2019; Yin and Schnoor, 2022). Transgelin-2 activation is responsible for the dephosphorylation of MYPT1, leading to the activation of MLC phosphatase (MacDonald and Walsh, 2018; Yin et al., 2018). Subsequently, the activated MLC phosphatase inhibits dephosphorylating MLC, resulting in airway smooth muscle cell relaxation (Yin et al., 2018; Camoretti-Mercado and Lockey, 2021b). In addition to TSG12, the ligand of transgelin-2 (i.e., metallothionein-2) also relaxes airway smooth muscle when administrated before ovalbumin challenge (Yin et al., 2018). The interaction of transgelin-2 with metallothionein-2 mediates this relaxation effect (Yin et al., 2018). Based on our results, metallothionein-2 and other transgelin-2 agonists may have therapeutic effects on asthma. Therefore, our findings provide more experimental evidence and clues of new strategies for asthma treatment.

### AHR Is Different From Asthmatic Inflammation

AHR is characterized by excessive airway contraction in responses to various nonallergenic stimuli, such as Mch (Coates et al., 2017; Alessandrini et al., 2020). Mch directly interacts with muscarinic receptors on airway smooth muscle cells and results in airway contraction (Coates et al., 2017). Previous studies evaluated the cell counts of neutrophils and eosinophils of sputum induction before and after Mch challenge in stable asthmatic patients, showing no significant differences (neutrophils: 40.43 ± 6.35% vs. 44.11 ± 6.33%; eosinophils: 8.43 ± 3.32% vs. 9.58 ± 3.94%) (Spanevello et al., 1999). It is reported that the mean percentages of macrophages, neutrophils, eosinophils, and lymphocytes were similar after Mch (64, 26, 7, and 1%) and hypertonic saline (67, 26, 6, and 1%) challenge in patients with and without asthma (Belda et al., 2005). The hypertonic saline has been proved to have no effect on cell counts of sputum in healthy and asthmatic patients (Belda et al., 2005). Mch interacts with muscarinic receptors to induce AHR while inflammation cells interact with pattern recognition receptors and then mediate NF-κB to trigger AHR (Coates et al., 2017; Duan et al., 2022). We tested bronchoalveolar lavage fluid in an ovalbumin-induced asthma model, showing no effects on inflammatory cells after Mch inhalation (Supplementary Figure S3). Consistent with the previous studies, no significant differences in lung tissue inflammation were observed among groups after Mch administration.

### AHR Is Significant for Asthma and Current Treatments

AHR is one of the most important pathological features of asthma (Cheng et al., 2022). The clinical treatments for treating acute airway contraction of AHR include β_2_-agonists and muscarinic antagonists (Wendell et al., 2020). However, evidence showed that the administration of β_2_-agonists such as terbutaline induced receptor desensitization and lost its efficacy (Fogli et al., 2013). In addition, significant side effects, such as palpitation and cramps, further lead to the limitation of clinical application (Mansur et al., 2014). The high variability of response to muscarinic antagonists (such as tiotropium) is also the main obstacle in its clinical application (Ora et al., 2021). Our study showed that 1/1000 doses of TGS12 (100 ng/kg) can gain similar effects on reducing pulmonary resistance compared to TB (100 μg/kg). Therefore, transgelin-2 agonist may provide a new clue for drug development in treating the bronchial contraction of asthma.

### Role of Transgelin-2 in Airway Smooth Muscle Contraction

The qRT-PCR results showed that the expression of transgelin-2 was significantly increased when TSG12 inhalation both before and during AHR occurrence. As mentioned earlier, transgelin-2 is an actin-binding protein, which participates in the contraction of cells (Kim et al., 2021). A previous study showed that the expression of transgelin-2 increased when extracellular signal-regulated kinase interacted with transgelin-2 at serine 145 residue and therefore amplified the regulatory signals of cell proliferation (Sun et al., 2018). Transgelin-2 also plays an important role in mediating membrane–cytoskeleton interactions (Yin and Schnoor, 2022). Therefore, the increased transgelin-2 expression may facilitate the signal transduction process in regulating the contraction and relaxation of airway smooth muscle cells. In a short summary, our results suggest that the administration of TSG12 may help in relaxing airway smooth muscle by increasing the expression of transgelin-2.

### Role of MLC in Airway Smooth Muscle Contraction

The expression of MLC in our study has the same trends with transgelin-2. MLC is a subunit of myosin mediating interaction between actin and myosin filaments to regulate smooth muscle contraction (Kitazawa et al., 2021). MLC has many isoforms with various tissue distributions, including airway smooth muscle cells (Andruchov et al., 2006; Zhou DD. et al., 2020). It is reported that MLC9 and MLC12 are new functional ligands for CD69 and may contribute to asthma pathogenesis (Hayashizaki et al., 2016). The increased expression of MLC is also closely related to the faster rate of airway smooth muscle shortening (Léguillette et al., 2009). In general, our result of MLC also suggested that the specific myosin expression may play an important role in AHR.

However, there are some limitations in our study. The previous study showed the phosphorylation of transgelin-2 downstream proteins plays an important role in airway contraction, while the phosphorylation changes have not been observed in the study. In addition, the mouse model of AHR has been used in our study, showing both the preventive and therapeutic role of TSG12 in regulating the contraction and relaxation of airway smooth muscle. Further studies using airway inflammation models are needed to determine how TSG12 regulates inflammatory cells and downstream targets in different administration times.

In summary, our study shows that the transgelin-2 agonist effectively reduces pulmonary resistance when TSG12 inhalation occurred both before and during AHR, suggesting the effect may relate to the increased gene expression of transgelin-2 and MLC.

## Data Availability

The datasets presented in this study can be found in online repositories. The names of the repository/repositories and accession number(s) can be found in the article/Supplementary Material.

## Abbreviations

AHR, airway hyperresponsiveness; Cdyn, dynamic compliance; Mch, methacholine; MLC, myosin light chain; MYPT1, myosin phosphatase target subunit-1; RL, pulmonary resistance; TB, terbutaline.
