# Supplementary material for: The Effects of a Transgelin-2 Agonist Administered at Different Times in a Mouse Model of Airway Hyperresponsiveness
Source: Front Pharmacol. 2022 Jun 16;13:873612. doi: 10.3389/fphar.2022.873612 (PMC9243334; doi:10.3389/fphar.2022.873612)
Supplement: Supplementary file 1 [file DataSheet1.PDF]

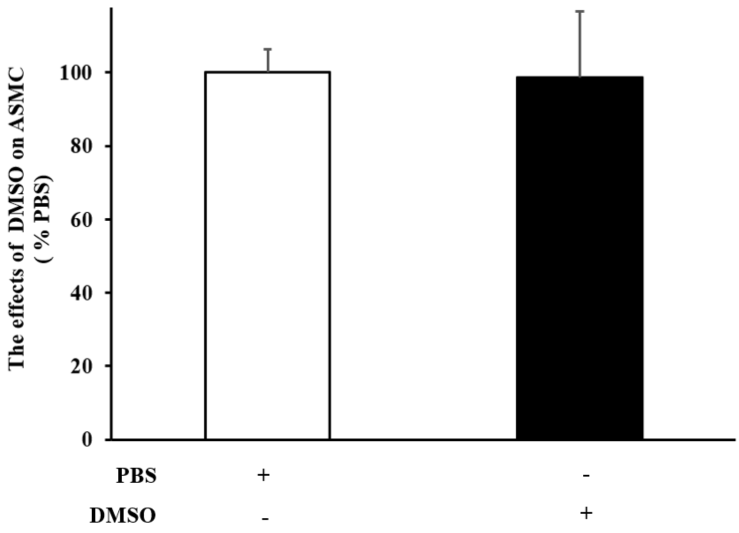

Figure S1. The comparison of effects of DMSO and PBS on airway smooth muscle cells. There was no difference between DMSO and PBS on airway smooth muscle cell Data were presented as the mean ± SEM, N=4, P<0.05 was considered statistically significant. **Abbreviation:** ASMC, airway smooth muscle cells; PBS, phosphate buffered saline; DMSO, dimethyl sulfoxide;

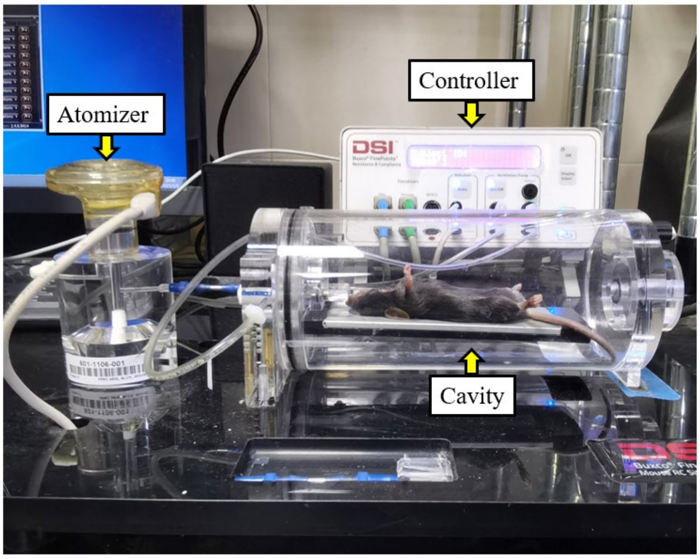

Figure S2. The photo of pulmonary function measurement in mice using the Resistance and Compliance System

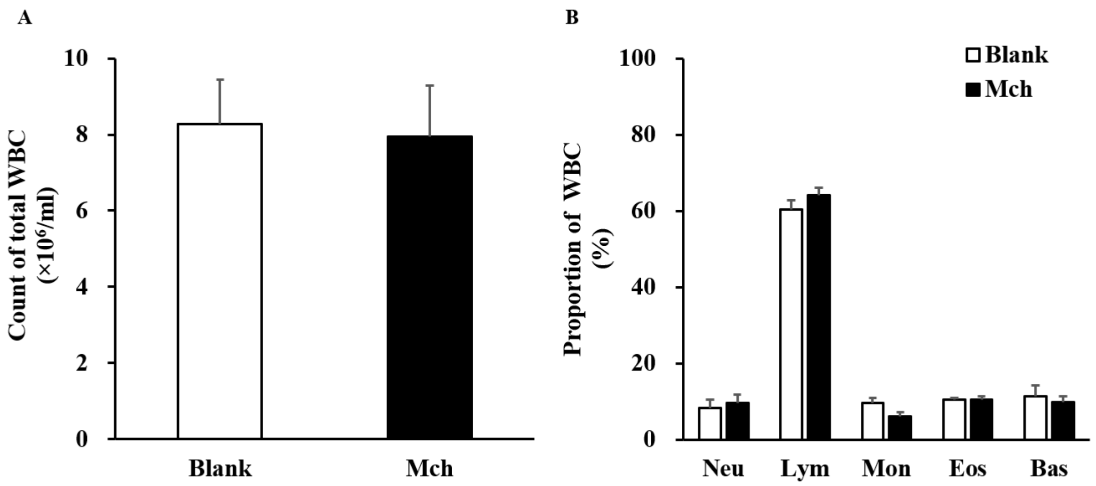

Figure S3. Inflammatory cell changes after methacholine inhalation in ovalbumin-induced asthma model. (A) There was no significance difference in the count of total WBC after Mch inhalation. **(B)** There was no significance difference in the proportion of WBC between different groups. Data were presented as the mean ± SEM, N=6, $P < 0.05$ was considered statistically significant. **Abbreviation:** Mch, methacholine; WBC, white blood cell; Neu, neutrophil; Lym, lymphocyte; Mon, monocyte; Eso, eosinophil; Bas, basophil.
